# Progress in the face of cuts: a qualitative Nigerian case study of maintaining progress towards universal health coverage after losing donor assistance

**DOI:** 10.1093/heapol/czab051

**Published:** 2021-05-04

**Authors:** Uche Shalom Obi, Osondu Ogbuoji, Wenhui Mao, Minahil Shahid, Obinna Onwujekwe, Gavin Yamey

**Affiliations:** Health Policy Research Group, College of Medicine, University of Nigeria, UNTH Road, Enugu State 400001, Nigeria; Department of Community Medicine, University of Nigeria Teaching Hospital, P M B 01129, Enugu State, Nigeria; The Center for Policy Impact in Global Health, Duke Global Health Institute, Duke University, 310 Trent Drive, Durham, NC 27708, USA; The Center for Policy Impact in Global Health, Duke Global Health Institute, Duke University, 310 Trent Drive, Durham, NC 27708, USA; Health Policy Research Group, College of Medicine, University of Nigeria, UNTH Road, Enugu State 400001, Nigeria; Department of Health Administration and Management, College of Medicine, University of Nigeria, Enugu Campus, Enugu, Nigeria; Department of Community Medicine, University of Nigeria Teaching Hospital, P M B 01129, Enugu State, Nigeria

**Keywords:** Development assistance for health, transition, Nigeria, UHC

## Abstract

In the coming years, about a dozen middle-income countries are excepted to transition out of development assistance for health (DAH) based on their economic growth. This anticipated loss of external funds at a time when there is a need for accelerated progress towards universal health coverage (UHC) is a source of concern. Evaluating country readiness for transition towards country ownership of health programmes is a crucial step in making progress towards UHC. We used in-depth interviews to explore: (1) the preparedness of the Nigerian health system to transition out of DAH, (2) transition policies and strategies that are in place in Nigeria, (3) the road map for the implementation of these policies and (4) challenges and recommendations for making progress on such policies. We applied Vogus and Graff’s expanded transition readiness framework within the Nigerian context to synthesize preparedness plans, gaps, challenges and stakeholders’ recommendations for sustaining the gains of donor-funded programmes and reaching UHC. Some steps have been taken to integrate and institutionalize service delivery processes toward sustainable immunization and responsive primary healthcare in line with UHC. There are ongoing discussions on integrating human immunodeficiency virus (HIV) services with other services and the possibility of covering HIV services under the National Health Insurance Scheme (NHIS). We identified more transition preparedness plans within immunization programme compared with HIV programme. However, we identified gaps in all the nine components of the framework that must be filled to be able to sustain gains and make significant progress towards country ownership and UHC. Nigeria needs to focus on building the overall health system by identifying systematic gaps instead of continuing to invest in parallel programmes. Programmes need to be consolidated within the overall health system, health financing priorities and policies. A comprehensive and functional structure will provide continuity even in the event of decreasing external funds or donor exits.

**Key messages**

- Nigeria’s health system does not have well-developed preparedness plans for transitioning out of development assistance for immunization and HIV programmes.
- There are policies and strategies in place to enable Nigeria to transition smoothly from such assistance and make progress towards universal health coverage (UHC); however, the implementation of these policies and strategies is hindered by systemic inefficiencies and poor leadership.
- The National Health Act and the Basic Healthcare Provision Fund are major health service and financing policies to protect poor populations, but their progress has been hindered by multiple challenges and bottlenecks.
- Increased fiscal space for, and budgetary allocation to, health is crucial to make progress but cannot be achieved without good governance and accountability.

## Introduction

Development assistance for health (DAH) continues to be an important source of health funding in low- and middle-income countries, including Nigeria (Bendavid *et al.*, 2017; Kotsadam *et al.*, 2018). The turn of the new millennium witnessed a dramatic growth in DAH, with a rapid annual growth rate of 11.3% in the first decade from 2000 to 2009 (the so-called ‘golden era’ for global health), followed by a decline in the annual growth rate to 1.2%, from 2010 to 2015 (Morrison, 2012; Dieleman *et al.*, 2016). In addition to the slowing growth rate in DAH, many countries are expected to graduate (transition) from receiving DAH, based on the eligibility criteria for receiving DAH (McDade *et al.*, 2020).

Different donors use a variety of eligibility criteria for determining which countries received DAH and, consequently, when these countries would transition away from such aid. The multilateral agencies such as the Global Fund to Fight AIDS (acquired immune deficiency syndrome), TB (tuberculosis) and Malaria (the Global Fund) and Gavi, the Vaccine Alliance (GAVI), use gross national income (GNI) per capita either solely or as part of their eligibility criteria (Ottersen *et al.*, 2017). Countries that cross a certain GNI per capita threshold are becoming ineligible for funding and are transitioning out of DAH. However, an increase in GNI per capita does not necessarily translate to equitable distribution of resources or to a reduction of disease burden, and so a country that has transitioned out of DAH may face the prospects of dealing with these persisting health challenges (Kanbur and Sumner, 2012). As countries strive to achieve UHC, sustaining vital health investments and benefits for their populations in areas such as human immunodeficiency virus (HIV) and immunization is crucial for making meaningful progress towards UHC (Resch and Hecht, 2018).

In Nigeria, DAH constitutes about 8.6% of total health spending (Chang *et al.*, 2019). Major disease control interventions such as HIV/AIDS, TB, malaria and vaccination programmes—including polio eradication—are mostly funded by a narrow group of external funders, including the Global Fund, the U.S. President’s Emergency Plan for AIDS Relief (PEPFAR), GAVI, the UK’s Department for International Development (DFID) and other bilateral funders (Bendavid *et al.*, 2012; Dieleman *et al.*, 2014).

The Global Fund uses a combination of income classification and disease burden for eligibility criteria. While Nigeria is still eligible for funding from the Global Fund due to its high disease burden, it needs to pay close attention to the possibility of losing external funds, given the overall decline in the growth rate of DAH. However, GAVI bases its eligibility criteria on a country’s GNI per capita; countries with a previous three-year average GNI per capita of less than or equal to US$1580 are eligible for support (GAVI, 2017). Nigeria’s three-year average GNI per capita (2016–2018) for 2020 eligibility is US$2163 (GAVI, 2019). This three-year average is well above the eligibility threshold, and so GAVI was set to transition out of providing financial support to Nigeria in 2021. However, given that Nigeria has one of the lowest vaccination rates in the world, GAVI extended its support to 2028 (Adepoju, 2018). This extension, coupled with the low rate of basic immunisation (31%) (Federal Ministry of Health [FMOH] NDHS, 2018) suggest that Nigeria was unprepared to take over and sustain immunisation for its population.

**Figure 1.**
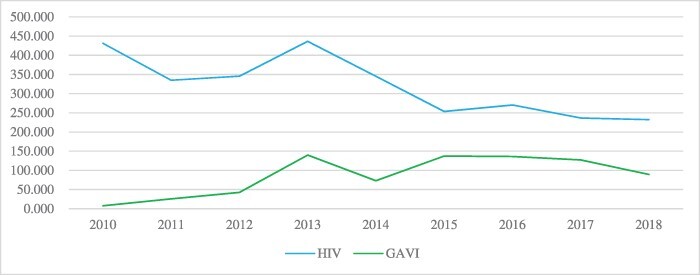
Trend of gross disbursement of Official Development Assistance from all channels for HIV and immunization programs in Nigeria Source of data - Creditor Reporting System (CRS)

**Figure 2.**
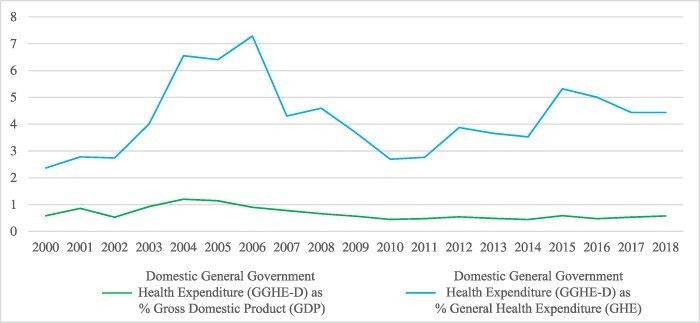
Trend of Nigeria’s Domestic General Government Health Expenditure (GGHE-D). Source from Global Health Expenditure Database

Despite being a lower middle-income country, Nigeria stands out as much unprepared to transition out of DAH. The country still has the third largest HIV epidemic in the world and a relatively high HIV incidence rate (WHO, 2017). The decline in donor funding has been reflected in weaker service delivery, such as requiring patients to pay out of pocket for certain tests that were initially free (Figure 1) (Moszynski, 2010). Nigeria also has poor maternal and child health indices: only 31% coverage of basic immunization for children aged 12–23 mo; an under-five mortality rate of 132 deaths per 1000 live births; and a maternal mortality ratio (MMR) of 512 per 100 000 live births (The Federal Republic of Nigeria, 2019). The Nigerian health system continues to suffer as a result of persistently low levels of domestic financing for health—an average of 14.5% government health spending per total health spending in 2016, showing very poor government commitment to health (Figure 2) (Chang *et al.*, 2019).

A comparative analysis of past cohort of graduated countries (2010 and 2015) and upcoming cohort of graduating countries indicated that Nigeria will be vulnerable to health shocks after transition (Gavin Yamey *et al.*, 2018, p. 15). This analysis showed that, on average, ‘countries that graduated between 2010 and 2015 period had stronger capacity to manage the donor transition than countries that are due to transition out of multilateral assistance in coming years. The upcoming cohort seems to have, on average, lower per capita income, greater indebtedness, weaker capacity to efficiently use public resources, more limited and less-effective health systems, weaker governance and public institutions and greater inequality.’ Thus, the countries that are facing transition do appear to be more vulnerable to setbacks in the coming years when compared with the past graduates.

Apart from being in the category of ‘vulnerable upcoming cohort of countries’, Nigeria also recorded the highest maternal mortality ratio (MMR) among these next cohorts of graduating countries. The possibility of losing funds at a time when there is need for accelerated progress towards UHC is a source of concern, as this will further widen the existing gap. More so, considering the low government funding for health in Nigeria (14.5% of the total health expenditure), this impending financial gap will most likely shift to out-of-pocket spending, which constitutes 75.2% of the THE (Chang *et al.*, 2019). This will be catastrophic, and will drive more of the vulnerable population below poverty line. Given these challenges, Nigeria faces several obstacles in making progress towards UHC.

Experience from countries that have transitioned out of DAH, such as South Africa, Botswana, Namibia and Eastern Caribbean countries, show that transition must be well-planned and managed to be able to sustain health gains (Vogus and Graff, 2015; Burrows *et al.*, 2016). Nigeria will need a comprehensive plan to drive country ownership of donor-funded programmes to retain the gains achieved from these programmes, and to avoid further declines or interruptions in service delivery.

With changes in DAH and impending transitions, the need for sustainable solutions has put transition high on the global health agenda (Ogbuoji and Yamey, 2018; Resch and Hecht, 2018; Bliss and Peck, 2019). Developing such sustainable solutions require an understanding of what stakeholders in Nigeria think about the possibility of losing funds, and the plans in place to make progress towards UHC when the external funds for HIV and immunization decrease or end. This study explored the perspectives of stakeholders on: (1) the preparedness of the Nigerian health system for responsible transition, (2) whether policies and strategies are in place for implementing such transition, (3) the potential challenges ahead on transitioning to domestic funding of health and (4) how Nigeria can make progress towards UHC.

## Methods

### Study design and setting

This study used qualitative research methods to explore stakeholders’ perception of transition from external funding to domestic funding for priority public health services in Nigeria. The tracer diseases were HIV and immunisation. Individual in-depth interviews were conducted in person or by phone with key informants (KIs) in Nigeria (domestic policy actors) and Geneva (international policy actors), from August to November 2019.

A semi-structured questionnaire was used to explore KIs’ views on: (1) the preparedness of the Nigerian health system for the upcoming transition away from DAH, (2) the underlying contextual barriers to responsible transition (3) policies and strategies that relate to sustainability of gains and progress towards achieving UHC, and (4) recommendations for progress and challenges to implementation of best practices.

### Study sample

The study population consisted of key stakeholders in HIV control, immunisation, UHC and NHIS programmes in Nigeria, both at the national and subnational levels, as well as external donors. Individuals were eligible to participate if they were representatives of donors, programme directors, programme officers, general managers, executive secretaries, implementing partners, desk officers, policymakers and experts involved in HIV control, immunization, UHC and NHIS programmes in Nigeria. HIV and immunization are two disease priority areas in Nigeria that are mostly funded through external aid.

At the national level, respondents were recruited from the FMOH, the National Primary Health Care Development Agency (NPHCDA), National Agency for Control of AIDS, UHC programme and NHIS. At the subnational level, respondents were recruited from the State Ministry of Health, State Primary Health Care Development Agency, State UHC programme and State Health Insurance Scheme. Stakeholders were selected based on their expertise and experience in health financing and health systems, and based on their roles in HIV control, immunization, UHC and NHIS programmes in Nigeria. Key-role players in HIV control, immunization, UHC and NHIS, both at the national and state levels were selected. Stakeholders also included health financing and health system experts. Key-role players were purposively selected to reflect the nuances of donor–recipient and federal-/state-level perspectives. The selection also aimed to achieve representation of diverse perspectives and social stratifiers such as gender and professional category.

State or subnational interviews were conducted in Enugu state. Enugu state was selected for the subnational interviews as a result of preliminary findings, suggesting a decline in HIV funding as reflected in severe shortage of HIV testing commodities (Ogbuabor, 2020). Respondents with less than 2 yr of experience in their current role were considered not sufficiently experienced and were excluded from the study. The size of the sample was initially set at 12 to 16 in-depth interviews, but we continued conducting interviews until we reached data saturation. A total of 17 respondents were interviewed (Table 1). One of the proposed respondents (a donor) refused to be interviewed for uncertain reasons.

**Table 1.** Table of respondents

Key informant (KI) number	Gender	Level of expertise	Role
KI 1	Male	National	Programme director
KI 2	Male	National	Healthcare financing consultant
KI 3	Female	National	Healthcare financing consultant
KI 4	Female	National	General manager
KI 5	Male	Sub-national	Policymaker
KI 6	Male	National	Policymaker
KI 7	Female	Sub-national	Policymaker
KI 8	Male	National	Health system consultant
KI 9	Male	National	Healthcare financing consultant
KI 10	Female	Sub-national	Programme director
KI 11	Male	National	Country coordinator
KI 12	Male	National	Healthcare financing consultant
KI 13	Male	National	Executive secretary
KI 14	Male	Sub-national	Executive secretary
KI 15	Male	National	Desk officer
KI 16	Female	National	Health system consultant
KI 17	Female	International	Country director

### Interview procedures

We emailed participants the information sheet that contained a brief description of the purpose of the study, the participant’s role in the study and ethical considerations. Participants who were willing to be interviewed were asked to suggest a convenient time and place and medium (example: landline, Skype and mobile phone) for the interview. The interviews were conducted in person (12 interviews) and by telephone (five interviews). A semi-structured interview guide was used to facilitate the discussion. Each of the interviews lasted an average of 46 min (range 28–88 min). All interviews were conducted in English and were audio-recorded with the consent of the participants.

A total of 17 respondents were interviewed (11 males and six females). Ten national-level and six subnational interviews were conducted with stakeholders in Nigeria; and one donor interview was conducted with a stakeholder in Geneva.

### Data analysis

Data analysis proceeded simultaneously with data collection, and emerging findings informed deeper enquiries in subsequent interviews. The audio-recorded interviews from the study were transcribed verbatim, and a thorough accuracy check was done on two randomly selected transcripts to validate the accuracy of the transcription process. Two interviews that were deemed to be particularly rich in information were used to generate the initial code book. The code book consisted of structural codes that were informed by the interview guide and inductive thematic codes that emerged in the interviews. All transcripts were uploaded in NVivo 12 and were coded by two different members of the team. The major themes used in coding responses were perspectives of respondents on donor funds, perspectives of respondents on sustainability and responsible transition, transition preparedness, key steps for making progress and challenges to making progress.

### Framework for data analysis

**Figure 3.**
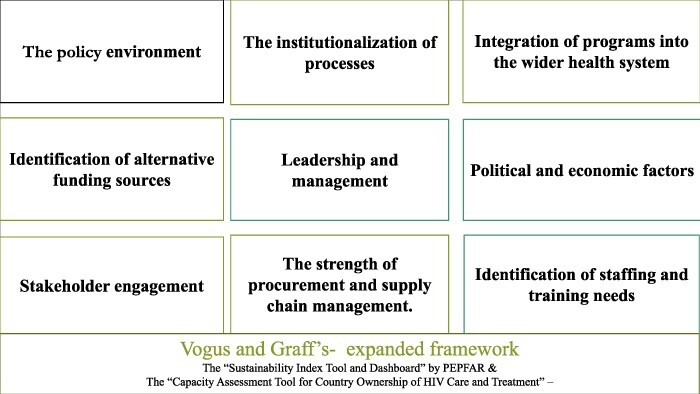
Vogus and Graff’s expanded framework for evaluating country readiness for transition of donor-funded health programmes to domestic ownership (Vogus and Graff, 2015)

There are several transition tools and frameworks in the literature for assessing the sustainability of donor-funded programmes For example, the ‘Sustainability Index tool and Dashboard’ released by PEPFAR in 2015 focuses on existing plans or road maps towards sustainability (PEPFAR, 2015), and captures various facets of successful ownership. PEPFAR’s ‘Capacity Assessment Tool for Country Ownership of HIV Care and Treatment’ provides a framework for assessing country capacity for more focused planning, organization and management of HIV programmes (PEPFAR, 2013). Vogus and Graff built and expanded on these two tools to develop a transition-readiness framework that has nine key areas for evaluating country readiness for transition to ownership (Figure 3).

The Vogus and Graff framework expands on existing transition readiness tools to capture more detailed components of preparedness based on the literature (Vogus and Graff, 2015). These nine key areas of the framework capture transition-readiness, and should be evaluated to determine country readiness for transition to ownership. We applied this expanded framework within the Nigerian context to evaluate stakeholders’ perspectives on preparedness and barriers to responsible transition and UHC. The framework asserts that there are nine key components that underlie country readiness for programme ownership: (1) policies that protect the rights of vulnerable populations, (2) institutionalization of processes that are integral to programmes to enable sustainability, (3) effective integration of programmes into existing administrative structures, (4) alternative sources of funding, (5) leadership and management capacity, (6) consequences of political and economic factors on health programming and outcomes, (7) stakeholder engagement, (8) procurement processes and (9) retention of qualified staff (see Figure 3). Furthermore, to underscore relevant areas of the health system that require careful consideration, we structured the stakeholders’ recommendation under the WHO health system building block.

## Results

In the following, we organize the emerging themes of the study into the nine categories in the Vogus and Graff framework (summarized in Table 2), followed by KIs’ recommendations for managing transition and making progress towards UHC.

**Table 2.** Summary of findings on the nine components of Vogus and Graff’s framework

Components of the framework	Summary of findings
Policy environment	There are many health policies and strategies; however, policy implementation is a major challenge. The National Health Act (NHA) and the Basic Healthcare Provision Fund (BHCPF) are the two major policies to protect the vulnerable population and make progress towards achieving UHC.
Institutionalization of the process	Visible steps have been taken to institutionalize service delivery processes toward sustainable immunization services and responsive primary healthcare (PHC) in line with UHC. There is a shift in the implementation approach within the HIV control programme to engage Nigeria more directly in programme implementation to enable sustainability of HIV services.
Integration of programmes into the wider health system	Some steps have been taken to integrate the immunization programme into PHC services and private services. There is ongoing discussion on integrating HIV services with other health services, and on the possibility of covering HIV services under the NHIS.
Identification of alternative funding processes	There is very little government funding for the HIV response. Major commitments have been made by the government to increase domestic funding of immunization. There are three health funding gaps in Nigeria:
Leadership and management	Leadership is laden with corruption, poor management processes and poor government commitment to health. The health system is overly dependent on donor funds, even in priority health areas such as HIV and immunization. Multiple health policies and commitments are made by the government without implementation. Accountability frameworks are developed and endorsed, but are rarely implemented. Coordinating the implementation of frameworks is a huge challenge. The health system lacks high-quality data for projections, proper management and planning. The dysfunctional structure of the Nigerian health system stands in the way of good management and proper coordination and hampers proper coordination across the multiple vertical programmes. The sharing of power across the three levels of government within the health system, with each level having some autonomy, makes the system difficult to negotiate.
Political and economic factors	Nigeria’s political environment is encumbered with political instability that results from changes in government tenure. With every change in government comes an abrupt end of health programmes instituted by the outgoing government, and the launch of new programmes by the incoming government. This lack of continuity in some health programmes wastes resources and constitutes a barrier to sustaining gains and making progress. It is challenging to persuade the government to allocate funds to health. Economic instability is a hindrance to transition of health programmes to domestic funding.
Stakeholder engagement	There are gaps in stakeholder engagement—grassroots actors are poorly engaged. Some respondents had concerns about poor stakeholder engagement in charting the course for the BHCPF.
Strength of procurement processes and supply	There are corrupt practices and inefficiencies in the supply chain. There are gaps in procurement processes.
Identification of staffing and training needs	There are gaps in human resources for health leading to poor retention of skilled workers. Most staff involved in HIV and immunization are supported by donor funds, and so withdrawal of funds will create even greater gaps and widen inequities.

### Policy environment

Stakeholders agreed that there are many health policies and strategies in place already, to close existing health gaps in Nigeria and make progress towards UHC, but implementation of these policies/strategies is a major challenge. They noted that the National Health Insurance (NHI) and the National Health Act (NHA) are the major policies to protect the vulnerable population and make progress towards UHC. The NHI aims to provide every Nigerian with a form of insurance, which could be national, social or private health insurance. It is intended to segment the market, such that every economic stratum is captured within the scheme.

*Oh, we have loads of well-written policies and strategies. There’s hardly anything we don’t have a policy and strategy on. Nigeria does not lack policies; the documents are there. They will engage consultants to draw them up but that is where it will end.* (National-level stakeholder, Healthcare financing consultant)

Some of the respondents elaborated on the NHA, stating that it was signed in 2014, but was not implemented until 2018. This act stipulates that at least 1% of the national consolidated revenue fund (CRF) should be transferred to the Basic Health Care Provision Fund (BHCPF) every year. (The BHCPF is a fund that was established under the National Health Act, as the principal funding vehicle for the Basic Minimum Package of Health Services, while serving to increase the fiscal space and overall financing to the health sector. It is expected that the attendant service upscale arising from application of this funding, would assist Nigeria achieve universal health coverage [UHC] (Federal Ministry of Health (FMOH) *et al*., 2016). Implementation of the BHCPF was decentralised to the state level, and states were mandated to set up State Health Insurance Schemes, and put in 100 million naira (approx. US$0.26 million) in the state account as counterpart funds, to be able to access the BHCPF. This mandate was put in place to prepare the states to administer the fund. Allocation of the fund is targeted to the most vulnerable population, and so state-specific poverty indices and other parameters will be used for allocating the funds. It is expected that comprehensive implementation of the NHA will be able to cushion the effects of donor transition.

### Institutionalization of processes

Five respondents agreed that Nigeria has taken some visible steps to institutionalize service delivery processes toward sustainable immunization services and responsive primary healthcare (PHC) in line with UHC. One key informant elaborated that following the extended accelerated transition period granted to Nigeria by GAVI, the NPHCDA developed a 10-yr strategy document on immunization and PHC. The National Emergency Routine Immunization Coordination Center (NERICC) was set up for implementing this strategic plan. So far, NERICC has been replicated in 18 prioritized poor-performing states as State Emergency Routine Immunization Coordination Centers. And at the local government level, NERICC has been replicated as the Local Emergency Routine Immunization Coordination Center. This replication is also expected to be operational at the ward level. These centers are established at the grassroots as point of service delivery processes.

*Now, what we are doing at the NPHCDA is that whatever we have at the national, we are currently making sure that we replicate them at the lower levels, we design program strategies and also coordinate and support the subnational levels to make sure they implement these programs. Let me give you an example, at the national level, we established National Emergency Routine Immunization Coordination Center. We made it mandatory for eighteen prioritized poor performing states to establish a similar structure as State Emergency Routine Immunization Coordination Center. We also made sure that this was established at the LGA and ward level.* (National-level stakeholder, Desk officer)

The KI 15 (national-level stakeholder) further stated that based on the experience from NERICC, another center was set up to tackle maternal and child health, called the National Emergency Maternal and Child Health Intervention Center (NEMCHIC). The NEMCHIC was also replicated at the state, local government and ward levels, and is expected to deliver maternal and child health interventions, including HIV services. Three key informants (KI 3, KI 13 and KI 16) highlighted the need to also institutionalize vaccine production in Nigeria, noting that this may be challenging due to lack of bioequivalence testing laboratories and local production of additives.

*We set up another centers (NEMCHIC) that are meant tackle all interventions related to women and children, including HIV/AIDS, malaria, and others. We are now rolling it out.* (National-level stakeholder, Desk officer)

Three respondents highlighted recent changes in the implementation approach for HIV programmes, a new approach focused on reprioritization and redeployment of funds in a more efficient manner. They described the changes as a shift towards a more structured way of operation, one that will also engage Nigeria more directly in programme implementation. Engaging Nigeria more directly in running the HIV programme will enable her to gain experience in the process, as well as build her capacity for sustainability.

*There’salready a shift towards enquiries on how the Nigerian government will implement the programme in such a way to make it easier to transition. In other words, thinking more realistically about how the host country government will be the one directly deploying the funds. That way, they can start to run the programmeand gain experience about how the programmes are run, and what investments are required to run the programme. You know, implementing the programmeby themselves to find cheaper ways of doing business*. (National-level stakeholder, Country director)

### Integration of programmes into the wider health system

Most KIs highlighted gaps in integration of donor programmes within the Nigerian health system as a barrier to making progress. They noted that while immunization has been more integrated into the public and private sectors, integration has been more challenging in the HIV response compared with the immunization response. Four KIs highlighted ongoing discussions about integrating HIV services with other services, and the possibility of covering HIV services under the NHIS. KI 15 (national-level stakeholder) also highlighted steps that the NPHCDA has taken in an attempt to integrate HIV and immunization services under PHC. He further noted that while immunization is more integrated into public health services, with some private-sector involvement, stigma is still a barrier to integration of HIV services with other health services.

*‘IntegratingHIV with other services has been a major challenge;however, opportunities are being explored right now regarding including HIV ultimately as part of the National Health Insurance Program. This appears to be an opportunity.*’ (Subnational-level stakeholder, General manager)

### Identification of alternative funding sources

Most key informants noted that Nigeria’s low level of domestic funding for health has been a long-term barrier to progress toward UHC. One key informant further described the health financing gaps in Nigeria as three dimensional. The first dimension is the current gap in domestic funding for health, even in the presence of support from various donors. The second dimension is the gap that is created as donors leave. The third dimension is the gap due to unpredictable economic factors. This KI noted that tackling this triple vacuum in health financing at the same time will be challenging. Respondents suggested that Nigeria needs to make significant domestic investments in health, in order to fill existing and impending gaps, and make substantial progress towards UHC.

While four KIs agreed that major domestic commitments have been made to fund immunization, five others noted that there is very little government funding for Nigeria’s HIV programme. Three KIs also noted that with competing priorities and the unstable economy, keeping to commitments and making further investments in health will constitute a challenge, considering the low priority given to health in Nigeria.

*[The] Nigerian government has underfunded health chronically over the years. Domestic funding is almost non-existent in HIV. Without the donors, I don’t know what would be the case.* (National-level stakeholder, General manager)

*It’s challenging to get the government to allocate money to health. I can tell you how we struggle to convince the parliament to allocate money to support the programme. And then after allocation, you also have to lobby the ministry of finance; in fact, sometimes you have to use contacts at the presidential villa to release the funds. It is really challenging.* (National-level stakeholder, Executive secretary)

### Leadership and management

Most KIs agreed that Nigerian leadership is laden with very poor government commitment, corruption and poor management processes. They noted that multiple policies and commitments are made without implementation, arguing that the government lacks commitment to health and is overly dependent on donor funds for financing priority health responses.

*One, I said that good leadership and political commitment is lacking—without that you can’t make progress. If you recall, sometime over ten years ago, we had what we called the* “*Abuja Declaration of 15% for health”[a target to allocate 15% of a national budget to health]; Nigeria has never gotten up to 10%.* National-level stakeholder, Healthcare financing consultant)

Six KIs highlighted corruption as barrier that must be minimized to be able to make progress. They stressed that the underlying problem is not just unavailability of domestic funds for health, but also, how the available funds are used or managed. Respondents further argued that there are major efficiency gains that could be harnessed if funds are utilized with more transparency and accountability. Two respondents also noted that there are developed and endorsed accountability frameworks that are not implemented, stating that, coordinating implementation of these frameworks has been a huge challenge.

*It is not about money but the system that will not just allow the programme to survive. Some will even tell you that the released money will go down into the politician’s pocket. The apprehension is real because the system is [neither] efficient nor effective to utilize the funds. Nigeria is not exactly the most transparent and accountable country; we can get more value for the health expenditure.* (National-level stakeholder, Executive secretary)

Nine out of 17 KIs noted that Nigeria lacks quality data for projections, proper management and planning. They noted that lack of reliable data with sufficient disaggregation of the population profile constrains effective planning and limits evidence-based decisions. Two KIs also noted that even the available data are mostly collected vertically by different programmes, creating inefficiencies within the system.

*[Nigeria has]asystem that does not have quality data for decisions and policy generation; a system that doesn’t follow through with planned programs to ensure full implementation.* (National-level stakeholder, Healthcare financing consultant)

Most KIs said that the dysfunctional structure of the Nigerian health system stands in the way of good management and proper coordination. They noted that this challenge has been long-standing because the PHC component of the system, which serves vulnerable populations, is placed at the lowest level with the least resources. They also noted that the fact that health is on the concurrent list (i.e. power is shared by the three levels of the government with each level having some autonomy) makes the system difficult to navigate. Thus, navigating through the independent levels of the government for effective implementation of reforms is challenging. This poor structure and extremely weak and dysfunctional system, said KIs, also means that multiple vertical programmes are being implemented without coordination. Lack of coordination of donor funds results in duplication of effort and inefficient use of available resources.

*So, the huge gap attracting foreign aid is a product of [an] extremely weak and dysfunctional system. It’s never the fund! It is about the dysfunctionality of the system. When you throw money into this kind of dysfunctionality, that fund too will follow the same route.* (National-level stakeholder, Programme director)

### Political and economic factors

Almost half of all KIs highlighted the consequences of political and economic factors in health outcomes and programming in Nigeria. They noted that the Nigerian political system suffers from political instability that arises with every change in government. Every new government ushers in a fresh start, i.e. new agendas and programmes, different from those of the preceding government. This instability, said the KIs, wastes expended resources and constitutes a barrier to sustaining gains and making progress towards UHC. Also, with the current economic instability in the country, it is very challenging to get the government to allocate funds to health.

*Government is not continuous as political changes still constitute a huge barrier. When any political group come[s] in, they start their own fresh agenda, trying to do things theirown way instead of following the track of the predecessors. Institutions have not matured in Africa, it is still a question of who or what party is in power. The leaders haven’t elevated themselves to that realm of intellectualism. They are still at the pedestal level of ‘we win election, we are in-charge for four years’*. (National-level stakeholder, Health system consultant)

### Stakeholder engagement

Seven KIs highlighted gaps in engagement of healthcare stakeholders as a barrier to progress, especially when it comes to grassroots actors. Three of the respondents particularly highlighted concerns about poor stakeholder engagement in charting the course for the BHCPF, noting disengagement of certain stakeholders. A national-level stakeholder further argued that there seems to be some form of division within the system due to multiple implementing organizations, with stakeholders wanting to get a share of the funds. A subnational-level stakeholder further buttressed poor engagement of stakeholders at the subnational levels of the healthcare system.

*I think that’s where the problem is. Citizens of Nigeria live in states, not in Abuja, so if we don’t get that tier of government very involved with this agreement early in these negotiations, we are not going to make progress. We need to change the way we do business and engage them. I have reservations with the Basic Heath Care Provision Fund. At some point, they started shutting people out because they felt threatened.* (National-level stakeholder, Executive secretary)

### Strength of procurement and supply chain management

Three KIs highlighted gaps in procurement processes, noting corrupt practices and inefficiencies in the supply chain for drugs, vaccines and commodities. They also highlighted a huge gap between procured and administered vaccines implying that some of the vaccines procured are not administered.

*But government prefers to give contracts to local contractors who will go and buy from the sales reps of the manufacturer. By the time they do this, the price is almost doubled. But when you have the money in a common purse, it doesn’t matter who owns the money. You can order straight from the manufacturers at a very cheap rate, and it will reach more people. But it means nobody is going to make money from it, but that is not what politicians want.* (National-level stakeholder, General manager)

### Identification of staffing and training needs

Five KIs noted gaps in human resources for health, particularly related to poor retention of skilled workers. Four respondents also raised concerns about placement of inexperienced staff in positions for which they were unqualified. They argued that most appointments are political rather than capacity-based. Two respondents also noted that most staff involved in HIV and immunization programmes are mostly supported by donor funds, and so withdrawal of these funds will create even greater gaps and widen inequities.

*People are sitting in positions to implement what they don’t have the capacity do. Not everyone who is in the position to lead an organization have the capacity to do so. So that’s part of the problem.* (National-level stakeholder, Health system consultant)

*Extremely weak and dysfunctional system; a system that is not merit or performance-based; a system that doesn’t focus on raising complementary level of human resources to address the specific health challenges.* (Sub-national-level stakeholder [Policy maker])

### Stakeholders’ key recommendations for making progress

Stakeholders gave several recommendations for ways to support a smooth transition from DAH and to make progress towards UHC. In the following, we structure stakeholders’ recommendations according to the components of the WHO’s health system building blocks framework (summarized in Table 3) (World Health Organization (WHO), 2007)

**Table 3.** Summary of stakeholders’ recommendation for making progress in transitioning from DAH and reaching UHC

Building block	Stakeholders’ recommendation
Leadership and governance	Intensive advocacy is needed to put transition preparedness on the agenda and to get all hands-on deck towards a robust transition plan. Coordination, management and oversight should be decentralized to the lower levels with commensurate resources and authority. Implementing good governance and accountability can help strengthen health systems and build strong institutions. Stakeholder inclusion at all levels down to the end users is necessary to make meaningful progress.
Financing	Increased fiscal space for, and budgetary allocation to, health are crucial to make progress. Greater efficiencies are needed in the health financing system. A joint funding basket for both domestic and external funds is desirable.
HMIS	A well-coordinated quality HMIS is essential to enable proper planning. Good-quality HMIS will enable effective planning, coordination and allocation of funds to priority areas.
Service delivery	A strong and robust PHC system will enable complete ownership of all forms of service provision at the grass roots, including HIV services. The BHCPF is a good start, but it needs to be followed to the letter with utmost transparency and accountability.
Medical products and technology	Efficiency in the procurement processes needs to be increased by elimination of bureaucracies that introduce avenues for mismanagement of available resources.
	Donor support should be directed towards capacity building for local production of vaccines with intensive deliberations on feasible solutions to the challenges of local production of vaccines.

### Leadership and governance

Four KIs suggested that intensive advocacy is needed to put transition preparedness on the agenda and to foster an ‘all-hands-on-deck’ approach towards a robust transition plan. They argued that FMOH and other stakeholders need to begin conversations to draw attention to the upcoming transition in HIV and immunization programmes, as well as other health programmes.

*We have to be intentional about it [transition]. There’s really nobody taking action. There is [a] need for more research on the subject matter of transition in Nigeria, there is [a] need to raise awareness about the future that is coming. There is [a] need for more engagement with the government to make it real.* (National-level stakeholder, Policy maker)

Three KIs argued that coordination, management and oversight should be decentralized to the lower levels with commensurate resources and authority; because implementation of the health response at the federal level is highly inefficient. The government, they argued, needs to begin to consider ways to restructure the health system so that funds are directed to areas of greatest need (the PHC level). Two KIs further recommended that donors and recipient country should work together to ensure sustainability plans are built in at the planning stage of the programme.

*We need to restructure the health system in such a way that funds can be directed to priority level, we need to revise the healthcare system state by state. The three levels of the healthcare system must come together in each state and have an all buy into the state health care plan, universal health care. Donor assistance will be more sustainable if implemented at state level. We may also begin to consider relieving the LGA of all functions apart from health and education.* (National-level stakeholder, General manager)

Most KIs noted that investing in good governance and accountability can help strengthen health systems and build strong institutions. The Nigerian government needs to rise beyond personalizing political offices or leadership, they argued, to focus more on building strong institutions that cannot be disrupted by the electoral process or changes in government. Such strong institutions can sustain health gains and make progress towards UHC.

*Good governance and strong institutions are necessary to change the entire directives of donor funding in the country. The government of Nigeria must be propelled to garner political will to pay more apt attention to healthcare, to connect the link between investment in health, productivity and economic development. This can be done by more intense advocacy and advocacy coalitions to the government to sensitize them to make more investment in health.* (National-level stakeholder, Country coordinator)

Five KIs recommended stakeholder inclusion at all levels, down to the end users as necessary, to make meaningful progress.

### Financing

Most KIs said that the government must take major decisions on domestic financing in order to manage transition and reach UHC. It is crucial, they argued, to begin conversations on the possible avenues to increase fiscal space and budgetary allocation to health. This is challenging and requires a lot of dialogue among the ministry of health, the ministry of finance and the ministry of budget and national planning, both at the federal and state levels.

Some KIs further argued that opportunities should be maximised to increase efficiency in the system, and that a joint funding basket for both domestic and external funds is desirable, which can be implemented at the state level.

*Some states have started looking at one single account for health; the idea is that all the donors financing health for the state put resources into a single account for implementing the state health plan.* (International-level stakeholder, Country director)

### Health Management Information System

Six KIs said that a well-coordinated high-quality HMIS is essential to enable proper planning. The government, they argued, cannot effectively take over and sustain the response to priority diseases without a good grasp on health management information to quantify health needs and available resources, noting that good-quality HMIS will enable effective planning, coordination and allocation of funds to priority areas.

*Let’s do proper planning. I am not absolutely sure that the government of Nigeria has a grasp of the totality of the amount of resources that are coming into the country for health. Unless they know that they cannot plan for the future, they cannot plan for a sustainable transition, because they first must know how much it is costing for the health response, because at some point, the government will have to take over responsibility.* (National-level stakeholder, Healthcare financing consultant)

### Service delivery

Eight KIs said that establishing a strong and robust primary healthcare system will enable complete domestic ownership of all forms of service provision at the grass roots, including HIV services. The BHCPF is a good start, they argued, but the government needs to ensure that implementation is followed up to the letter with utmost transparency and accountability.

### Medicine and technology

Three KIs argued that government and donors need to increase efficiency in the procurement processes by elimination of bureaucracies that introduce avenues for mismanagement of available resources. They also suggested that donors should support capacity building for local production of vaccines.

## Discussion

### Principal findings and their policy implications

Stakeholders perceive that Nigeria’s HIV and immunizations programmes are unprepared to transition from DAH to domestic resources. This is mostly because both programmes are currently heavily dependent on external assistance. Few steps have been taken by the country, mostly within the immunization programme, to prepare for transition from DAH. However, there are many gaps that need to be filled for Nigeria to safely transition from donor support for its health system and to reach UHC.

Within the policy environment, the federal government has taken steps towards improving primary healthcare by initiating implementation of the BHCPF and NHA. While some stakeholders are optimistic that the BHCPF is pro-poor and a good step towards provision of affordable healthcare for all, a critical look at the BHCPF shows that it cannot assure UHC for Nigerians (Onwujekwe *et al.*, 2018). In addition, providing 1% of the CRF for health is far from meeting the Abuja declaration of providing 15% of the annual budget to health (WHO, 2001), a long-standing promise that Nigeria has failed to keep after 20 years (Olakunde, 2012) (WHO, 2001) (Biegon, 2020). In a financial feasibility analysis of using the BHCPF to provide basic minimum maternal and child health benefit package in Nigeria, (Onwujekwe *et al.* (2016) showed that a minimum of 4% of CRF would be required to cover the target beneficiaries. The authors further recommended a re-evaluation of the level of funds allocated as BHCPF.

Through the NPHCDA and the NHIS, Nigeria has started implementing the BHCPF, taking steps to institutionalize service delivery processes and establish structures for implementation. These steps will enable structures for PHC delivery (including immunization) at all levels of healthcare (Onwujekwe *et al.*, 2018). Nigerian states have also been mandated to set up State Health Insurance as a prerequisite for assessing the BHCPF (Federal Republic of Nigeria, 2016). They are at different stages of implementation; however, full establishment and functionality of these structures need to be followed up intensively to evaluate progress and ensure quality service delivery.

Implementation of the BHCPF needs to be pursued earnestly with all stakeholders with particular attention to grassroot stakeholders. Our study found that there has not been proper stakeholder engagement in charting a course for the BHCPF—reports about shutting some actors out during plans for implementation could mean lack of transparency. Well-grounded stakeholder engagement is critical to progress. It is also crucial to follow up implementation of the BHCPF via the implementation structures (National Emergency Routine Immunization Coordination Center (NERICC) and National Emergency Maternal and Child Health Intervention Center (NEMCHIC)) being established at lower levels, to evaluate functionality and quality of service delivery.

Our study showed more gaps related to institutionalization and integration of HIV services within the health system compared with immunization. The shift in implementation approach within the HIV control programme is focused on country ownership. Respondents described the new approach as a more efficient way of programme implementation, with better engagement of recipient country to build country capacity for sustainability. This approach aligns with the 2005 Paris Declaration, the Accra Agenda for Action (OECD, 2005) and the Busan Partnership for Effective Development Cooperation (Organisation for Economic Co-operation and Development, 2012). These three documents underscored country ownership and sustainability of programmes requiring countries to have institutions to drive HIV interventions in order to make progress towards UHC.

Itiola and Agu argued that Nigeria has made some progress in relation to establishment of coordinating structures (Itiola and Agu, 2018). However, integration of HIV services with other services needs to be put into perspective. A comprehensive implementation structure that enables integration of vertical programmes for more efficient service delivery is crucial for making progress towards UHC (Global Health Initiative, 2012). Oleribe *et al.* (2014) have long called for ‘commonization’ of HIV services—that is, integrating HIV care into the existing fabric of the healthcare system for a sustainable and efficient healthcare system.

Increasing fiscal space in Nigeria is of pressing concern to stakeholders. Poor prioritization of health by the government, with its track record of failure to implement commitments, has been a long-term barrier to making progress (Cernuschi *et al.*, 2018). It is not surprising that Gavin Yamey *et al.* (2018) found that Nigeria is among the countries that are most vulnerable to setbacks when they transition from DAH in coming years (Gavin Yamey *et al.*, 2018). Yet, there is no clear plan for Nigeria to increase its fiscal space for health (Ichoku and Okoli, 2015).

Furthermore, stakeholders interviewed for this study argued that decades of poor financing for health cannot be separated from poor leadership and political commitment to health. The leadership environment is hindered by very poor commitment to health, corruption and poor management processes. Also, multiple policies and commitment are made without implementation. Uzochukwu *et al.* (2018) highlighted the depth of corruption in government as it relates to fund management. The authors stressed that health system corruption is a major barrier to successful implementation of the BHCPF.

Gaps in the political environment also create inefficiencies and lack of continuity in health programmes. With each new incoming government comes an abrupt end of ongoing health programmes started by the outgoing government and the launch of new ones. This disjointedness in health programmes has been witnessed over the years; the Midwife Service Scheme ended with government tenure (Ibe, 2015). Such abrupt change splits the country’s existence into different political tenures with lack of continuity in health and development programmes. This weakens the health system, making it more unresponsive and vulnerable to shocks. Already, the HIV programme is experiencing a redirection in flow of existing ODA to the prevailing Coronavirus Disease (COVID 19) pandemic response (Oladele *et al.*, 2020). This redirection could further destabilise the system.

The Nigerian health system is mostly unprepared to transition out of DAH and make progress towards UHC. This is predominantly due to policy implementation gaps and lack of initial transition plans at the onset of health programmes. To overcome the challenges and bottlenecks that hinder policy implementation and close these gaps, policymakers must give thoughtful consideration to policy implementation during the policy process. They must ensure that there are feasible road maps for apt implementation of pro-UHC policies.

Second, crucial steps must be taken to ensure that transition plans are factored into programme planning from the onset, and not as an addendum. Well-grounded health plan that puts transition in perspective is needed to foster sustainability and progress towards UHC at the time of transition. Nigeria must also endeavour to make political commitment to health—at least to live up to the 15% commitment of the annual budget to health, as promised in Abuja declaration of 2001 (WHO, 2001). This will enable the country to integrate healthcare programmes and build the overall health system to transition smoothly out of various donor programmes while making progress towards UHC.

It is crucial for the upcoming cohort of transitioning countries to have a robust transitioning plan in place to sustain gains of donor funds and make progress towards UHC. Transition plan should not be an afterthought but a well-incorporated aspect of health programme plan. Therefore, donors and recipient countries should ensure that transition plans are built into health programmes at the planning stage. Furthermore, it is not enough for upcoming transitioning countries to have pro-UHC policies; they should have pro-UHC policies as well as road maps for implementation, with broad stakeholder involvement. It is also crucial for such countries to give careful consideration to increasing their fiscal space for domestic funding for health, integration of health programmes and building the overall health system. This will engender sustainability and ensure progress towards UHC at the time of transition.

### Study limitations

The application of Vogus and Graff’s expanded framework within the Nigerian context enabled a robust synthesis of plans and gaps for making progress in all the components of the framework. However, the framework was not chosen prior to data collection, so the framework did not inform the data collection tool. Choosing the framework prior to data collection would have allowed a more in-depth exploration of the various components of transition preparedness of the framework.

Purposive sampling of experienced respondents that reflect the nuances of donor–recipient and state-/federal-level perspectives enabled well-informed and balanced perspective of the subject of enquiry. Exploring the subject to saturation allowed us to capture the perspectives of the KIs as much as possible. However, it is possible that non-response from one of the prospective donors could have introduced some bias into our study. It is also possible that a different group of KIs would have provided different opinions on the subject matter. It is unclear whether our findings have generalizability beyond Nigeria.

## Conclusion

To manage transition from DAH and make progress towards UHC, the Nigerian government needs to identify and address implementation gaps as well as systematic gaps in using domestic resources for financing critical health services. Policymakers must identify clear road maps for the implementation of the existing pro-UHC policies. Furthermore, funds should be redirected to building the overall system—consolidating and coordinating programmes and linking them into the overall health system, health financing priorities and policies. Instead of continuing to invest in parallel programmes, a comprehensive and functional structure for continuity—one that will be robust enough to withstand decreasing external funds or donor exits should be developed at the national and subnational levels as a matter of urgency. Donors and recipient countries should endeavour to have inbuilt transition plans to give direction to programme implementation approach and enable the institutionalization of service delivery processes for continuity.

## Abbreviations

AIDS acquired immune deficiency syndrome

BHCPF Basic Health Care Provision Fund

COVID coronavirus disease

CRF consolidated revenue fund

DAH Development Assistance for Health

DFID UK’s Department for International Development

GAVI Gavi, the Vaccine Alliance

GNI gross national income

HIV human immunodeficiency virus

HMIS Health Management Information System

KI key informant

MMR maternal mortality ratio

NHA National Health Act

NHI National Health Insurance

NHIS National Health Insurance Scheme

PEPFAR President’s Emergency Plan for AIDS Relief

SHIS State Health Insurance Scheme

TB tuberculosis

UHC universal health coverage

WHO World Health Organization
